# Performance analysis of channel estimation techniques for IRS assisted MIMO

**DOI:** 10.1038/s41598-023-40587-7

**Published:** 2023-08-21

**Authors:** Alelign Ewinetu Baye

**Affiliations:** Department of Electrical and Computer Engineering, Woldia University, Woldia, Ethiopia

**Keywords:** Electrical and electronic engineering, Mathematics and computing

## Abstract

The need for low latency and high data rates is increasing rapidly since the advent of wireless communication. The current fifth-generation (5G) networks are unable to fulfill the requirements of upcoming technologies. So, researchers are commencing their research beyond 5G. Terahertz (THz) frequency is one candidate to satisfy the large bandwidth requirement and intelligent reflecting surface (IRS) is incorporated to mitigate signal blockage which is the main problem for communication at high frequencies. Channel estimation is a process of identifying coefficients of the channel matrix. The compressive sensing technique is of great importance as it decreases the number of pilot symbols required for channel estimation. As mmWave and THz signals are naturally sparse applying a compressive sensing technique is reasonable. Unlike other papers, this paper considers the imperfect IRS elements, which is the real case, by varying the value of $\beta$ (amplitude perturbations). The channel estimation performance of the conventional least squares (LS), orthogonal matching pursuit (OMP) and Oracle is analyzed with respect to signal-to-noise ratio (SNR) and pilot length (T). Normalized mean square error (NMSE) and spectral efficiency (SE) are used as performance metrics and the OMP algorithm is found to perform better than LS even at a fewer number of pilot symbols.

## Introduction

As wireless communication progresses, systems are deployed which require high data rates and low latency and this need has been increasing and seems endless. For instance, between 2016 and 2021, a sevenfold increase is expected in mobile data and a threefold in video traffic^1^.

Nowadays, 5G networks come into existence. However, it is challenging for 5G networks to satisfy future requirements such as wireless charging, extremely low latency, performance uniformity in the coverage area, and immunity to jamming. To achieve these requirements, researchers have been undergoing beyond 5G. Artificial Intelligence (AI), smart wearable devices, Internet of Everything (IoE), and 3D mapping are among the technologies which hopefully are to be supported by beyond 5G networks^2,3^.

Unfortunately, the implementation of these technologies requires a very large bandwidth. THz frequency (0.1–10 THz), which is capable of providing an order of magnitude greater bandwidth than that of a millimeter wave, is a candidate attracting the attention of researchers. It is characterized by high directionality which in turn makes it robust for eavesdropping^4^.

Passive MIMO technologies called IRS also known as software-controlled metasurfaces^2^, are of great importance in avoiding blockage at a significantly low cost and power.

IRS is a physical meta-surface with many reflecting elements each of which is passive and can impose a phase shift on signals impinging them. The phase shifts are induced in such a way that the reflected signals add constructively or destructively to the desired receiver. The smart controller attached to the surface is responsible for arranging the reflecting elements^4–6^.

In conducting the channel estimation process, the concept of compressive sensing is applied. The Oracle (i.e., the benchmark), conventional LS, and OMP algorithms are used in the estimation process. A perfect reflection ($\beta =1$) is assumed for the conventional LS and Oracle. This assumption is made for the fact that the conventional LS is poor in estimation, so an imperfect assumption makes it worse. Furthermore, to show how powerful a technique OMP is in estimating sparse signals, the imperfect reflection assumption is applied for it. Again as Oracle is the benchmark algorithm, a perfect reflection assumption is considered to set it at its best performance.

Having the geometric channel model and using the poor scattering nature of the THz band, in^4^, the channel estimation problem is converted to a sparse recovery one. Then a compressed sensing (CS) technique called Iterative Atom Pruning Subspace Pursuit (IAP-SP) based channel estimation is applied to perform the task of channel estimation for a single-user MIMO. The authors in^7^ proposed the closed-form Least Squares Khatri–Rao Factorization (LSKRF) and an alternative Bi-linear Alternating Least Squares (BALS) channel estimation technique. Both methods are based on the tensor modeling approach of the received signal. An optimal Minimum Mean Square Error (MMSE) channel estimation algorithm is applied in^8^ to estimate the direct BS-to-user channel and the cascaded BS-to-IRS and IRS-to-user channel. In this channel estimation technique, the channel estimation process is divided into three phases. In each of the phases, the IRS elements are fed with optimal reflect beam-forming vectors, a result of which is that the optimal reflect beam-forming vectors are chosen by the optimal reflect IRS elements as the columns of a Discrete Fourier Transform (DFT) matrix. Furthermore, a closed-form expression for cascaded BS-to-IRS and IRS-to-user channels is provided by the DFT-MMSE technique depending on the prior information of large-scale fading statistics. In the paper^9^, the BS to Large Intelligent metasurface (LIM) and LIM to user channels are estimated separately using a two-stage algorithm with a sparse matrix factorization stage and a matrix completion stage. In the first stage Bilinear Generalized Approximate Message Passing (Bi-GAMP) algorithm is applied in recovering the LIM-user channel and the BS-LIM channel is estimated using joint bilinear factorization and matrix completion (JBF-MC) algorithm in the second stage. To reduce the pilot overhead the authors in^10^ exploit the double structure sparsity characteristic of the angular channels among the single-antenna users. For the channel estimation process the Double Structure Orthogonal Matching Pursuit algorithm (DS-OMP), which consists of two stages, is applied. The row-structured sparsity of cascaded channels helps to estimate the completely common row support in the first stage. In the second stage, the partially common column support is estimated by using the column sparsity characteristic of the cascaded channel. In^11^, mobile edge computing (MEC) assisted with IRS network architecture is suggusted to satisfy the low latency requirement of virtual reality (VR). The line-of-sight and non-line-of-sight statuses of VR users are identified through an algorithm which combines an online long-short term memory (LSTM) convolutional neural networks (CNN). A relationship between IRS beam pattern design and a two-dimensional finite impulse response filter design is establishe by^12^. And the problem was solved using a fast non-iterative algorithm. An efficient near-field IRS-assisted channel estimation scheme was proposed in^13^. The channel was estimated using polar-domain frequency-dependent RIS-assisted channel estimation (PF-RCE).

Mainly the main goal of this paper is to consider the non-ideal IRS case and showing its effects on channel estimation. Although there are many compresseive sensing based algorithms used to estimate a sparse signal, OMP algorithm is applied in this paper for its simplicity.

All the papers above consider a perfect IRS, ideal case, in the channel estimation process that they assumed as there is a perfect reflection of the impinging signals. But it is difficult to achieve such ideality in reality since there might be deficiencies in the manufacturing process of the metasurface. In addition, the metarials from which the metasurfaces are made have also their effect. Furthermore, environmental factors, such as, fog and dust affect the performance of IRS elements. Having this in mind, this paper shows the effect of the IRS by considering the non-ideal case and compares the results with the ideal (perfect reflection).

## Methods

### Compressive sensing

5G and beyond communication systems operate at high frequencies in the mmWave and THz ranges. Signal processing in these spectra requires a very high rate of sampling. This needs high-performance devices that may not be possibly manufactured or are very costly if so. This poses a need of finding a mechanism for representing data from many samples by taking only a few consisting of the gist of it. This is what compressive sensing tries to achieve^14^.

CS is to become a vital component in the next-generation wireless communication systems for the fact that many kinds of signals in wireless applications are sparse. Unlike Shannon’s theorem which depends on the highest frequency available for sampling, CS depends on the sparsity of signals. Saying another way, measurement signals proportional to the sparsity are required for reconstruction in the CS paradigm. CS comes with benefits: it saves storage, is energy efficient, lowers signal processing time, and solves problems that are said to be unsolvable in ordinary linear algebra^15–19^.

The entire process of CS consists of three steps:

- signal sparse representation;
- linear encoding and measurement collection;
- non-linear decoding (sparse recovery).

Among the greedy algorithms, OMP is one developed for sparse signal recovery^20,21^. Speed and ease of implementation are its merits^20^. Matching pursuit (MP) is applied in recovering a 1-sparse solution; whereas, OMP generalizes this for an s-sparse case. In OMP, the non-zero positions (supports) are estimated iteratively^22^.

An *m* dimensional measurement matrix ${{\textbf {y}}}$ is produced by the multiplication of an *n* dimensional s-sparse vector ${{\textbf {x}}}$ by matrix $\varvec{\Phi }$^23^ having a dimension of $m\times n$. That is:

1 $$\begin{aligned} {\textbf{y}} = \mathbf {\Phi } {\textbf{x}} \end{aligned}$$

where $\varvec{\Phi }$ is referred to as measurement matrix with columns $\varvec{\phi }_{1}, \dots , \varvec{\phi }_{n}$. The system represented in (1) is an under-determined problem since $n > m$ in most compressive sensing scenarios. The conventional inverse transform is unable to reconstruct the original signal ${{\textbf {x}}}$ from $\varvec{\Phi }$. However, having a priori information of sparsity and restriction on $\varvec{\Phi }$, ${{\textbf {x}}}$ can be reconstructed by solving the $\ell _{2}$-minimization problem^23^:

2 $$\begin{aligned} \min _{\varvec{x}} {\Vert \varvec{x} \Vert }_{2} \qquad \text {subject to} \quad \varvec{\Phi } \varvec{x} = \varvec{y} \end{aligned}$$

OMP follows a simple and intuitive principle; in each iteration, the most correlated columns of $\varvec{\Phi }$ with the residue are selected which is called identification, then the indices of this column are added to the list called augmentation, finally the residual is updated by removing the vestige columns from the measurements, this is called residual update. The OMP algorithm is shown in Table 1.

**Table 1:** OMP algorithm.

**Inputs:** measurement vector $\textbf{y}$, sparsity s, measurement matrix $\varvec{\phi }$	
**Output:** estimated channel $\hat{\textbf{H}_{c}}, \text {residual} {\textbf {r}}, \text {support} \Lambda$	
**Initialization:** $\varvec{r}_{0} = \textbf{y}, \Lambda _{0} = \emptyset \; \text {and iteration} \; t=1.$	
** for** $t<s$ **do**	
**Solving the optimization problem:**	
$$\lambda _{t} = \text {arg max}_{j=1 \dots d} |\langle \textbf{H}, \varvec{\phi }_{j} \rangle |$$	
**Augmentation of the support:**	
$$\Lambda _{t} = \Lambda _{t-1} \cup \{{\Lambda _{t}}\}$$	
**Estimation of the cascaded channel:**	
$$\hat{\textbf{H}_{c}} = \text {arg min}_{\textbf{H}} \Vert \textbf{y}-\varvec{\Phi }_{t} \textbf{H} \Vert _{2}$$	
**Update measurement vector and residual:**	
$$\textbf{y}_{t} = \varvec{\Phi }_{t} {\hat{\textbf{H}}_{c}}$$	
$$\varvec{r}_{t} = \textbf{y} - \textbf{y}_{t}$$	
** end for**	

### System model

**Figure 1:**
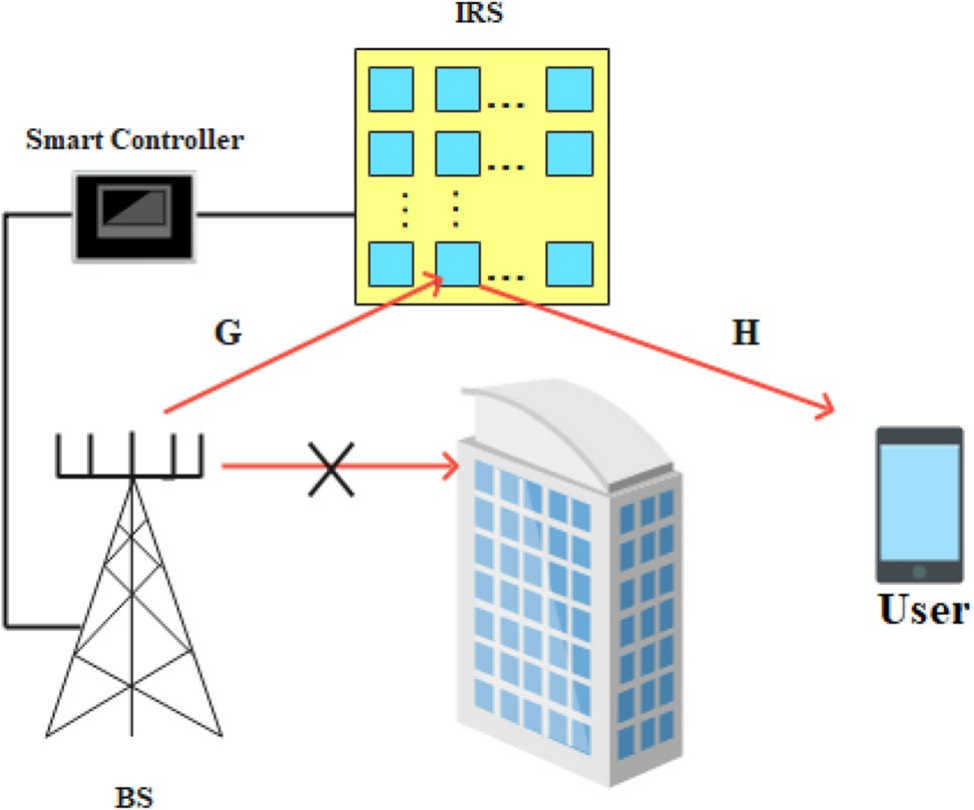
IRS assisted communication system.

Let’s consider a MIMO communication system assisted by IRS as shown in Fig. 1. A single mobile user equipped with $N_{r}$ antennas is considered. Additionally, the Line-of-Sight (LOS) path between the BS and the user is assumed blocked by local obstacles such as a building, and only the non-LOS path through the IRS is taken into account. The BS has $N_{t}$ number of antennas and the IRS consists of $N_{I}$ number of passive reflecting elements. ${\textbf{G}} \in {\mathbb {C}}^{N_{I} \times N_{t}}$ and ${\textbf{H}} \in {\mathbb {C}}^{N_{r} \times N_{I}}$ denote the channels from BS to IRS and IRS to user respectively.

A TDD mode of transmission is used so that the downlink channel can be estimated from the uplink channel due to the reciprocity property. In this research, the downlink transmission scenario is considered.

The phase shift matrix $\varvec{\Theta }$ of the IRS is mathematically expressed as^24^

3 $$\begin{aligned} \mathbf {\Theta }&= diag(\beta _{1}e^{j\theta _{1}} \; \dots \; \beta _{N_{I}}e^{j \theta _{N_{I}}}) \end{aligned}$$

where $\theta _{n} \in [0, 2\pi ]$ and $\beta _{n} \in [0, 1]$ represent the phase shift and amplitude respectively of the reflection coefficient associated with the n-th reflecting element.

The channels ${\textbf{G}} \in {\mathbb {C}}^{N_{I} \times N_{t}}$ and ${\textbf{H}} \in {\mathbb {C}}^{N_{r} \times N_{I}}$ are mathematically expressed as^10,24,25^

4 $$\begin{aligned} {\textbf{G}}&= \sqrt{\frac{N_{I} N_{t}}{P}}\sum _{p=1}^{P} \alpha _{p} \varvec{a}_{r} (\vartheta _{p},\gamma _{p}) \varvec{a}^{H}_{t}(\psi _{p},\phi _{p}) \end{aligned}$$

where $\alpha _{p}$ represents the complex gain of the p-th spatial path between the BS and IRS. $\vartheta _{p}$ and $\gamma _{p}$ are the azimuth and elevation angles of arrival (AoA) respectively at the IRS and $\psi _{p}$ and $\phi _{p}$ are the azimuth and elevation angles of departure (AoD) from the BS. In the same way, the channel ${\textbf{H}}$ is expressed as

5 $$\begin{aligned} {\textbf{H}}&= \sqrt{\frac{N_{I} N_{r}}{Q}} \sum _{q=1}^{Q}\beta _{q}\varvec{a}_{r}(\vartheta _{q},\gamma _{q} )\varvec{a}^{H}_{t}(\psi _{q},\phi _{q}) \end{aligned}$$

where $\beta _{q}$ represents the complex gain of the q-th spatial path between the IRS and a user. $\vartheta _{q}$ and $\gamma _{q}$ are respectively the azimuth and elevation angles of arrival (AoA) and $\psi _{q},\phi _{q}$ are the azimuth and elevation angles of departure (AoD). Furthermore, $\varvec{a}_{r}$ and $\varvec{a}_{t}$ represent the receive and transmit array steering vectors respectively. Suppose the IRS is $N_{I,x} \times N_{I,y}$ Uniform Planar Array (UPA)

6 $$\begin{aligned} \varvec{a}_{r} ( \vartheta _{p},\gamma _{p} )&= \varvec{a}_{x} (u) \otimes \varvec{a}_{y} (v) \end{aligned}$$

7 $$\begin{aligned} u&= 2 \pi d \frac{cos(\gamma _{p})}{\lambda } \end{aligned}$$

8 $$\begin{aligned} v&= 2 \pi d \frac{sin(\gamma _{p}) cos(\vartheta _{p})}{\lambda } \end{aligned}$$

d and $\lambda$ represent the antenna spacing and signal wavelength respectively. And $\otimes$ is the Kronecker product.

9 $$\begin{aligned} \varvec{a}_{x}(u)&= \frac{1}{\sqrt{N_{I,x}}} [1 \; e^{ju} \; \dots \; e^{j(N_{I,x} - 1)u}]^{T} \end{aligned}$$

10 $$\begin{aligned} \varvec{a}_{y}(v)&= \frac{1}{\sqrt{N_{I,y}}} [1 \; e^{jv} \; \dots \; e^{j(N_{I,y} - 1)v}]^{T} \end{aligned}$$

As the high-frequency channels have sparse scattering nature, the propagation paths are small in number compared to the dimension of the channel matrix. So, the channel ${\textbf{G}}$ can be written as^10,24^

11 $$\begin{aligned} {\textbf{G}}&= ({\textbf{F}}_{x} \otimes {\textbf{F}}_{y}) \mathbf {\Sigma } {\textbf{F}}^{H}_{L} \nonumber \\&= {\textbf{F}}_{P} \mathbf {\Sigma } {\textbf{F}}^{H}_{L} \end{aligned}$$

where ${\textbf{F}}_{L} \in {\mathbb {C}}^{N_{t} \times N_{tG}}$ is an over complete matrix $(N_{tG} \ge N_{t})$ and each of its columns has a form $\varvec{a}_{t} (\phi _{p})$, with $\phi _{p}$ chosen from a pre-discretized grid, ${\textbf{F}}_{x} \in {\mathbb {C}}^{N_{Ix} \times N_{IG,x}} ({\textbf{F}}_{y} \in {\mathbb {C}}^{N_{Iy} \times N_{IG,y}})$ is similarly defined with each of its columns having a form of $\varvec{a}_{x}(u) (\varvec{a}_{y}(v))$ and *u*(*v*) are from a pre-discretized grid, $\mathbf {\Sigma } \in {\mathbb {C}}^{N_{IG} \times N_{tG}}$ is a sparse matrix with P non-zero entries corresponding to the channel path gains ${\alpha _{p}}$, in which $N_{IG} = N_{IG,x} \times N_{IG,y}$. The true AoA and AoD are assumed for simplicity to lie on the discretized grid. In a similar fashion the channel ${\textbf{H}}$ is written as

12 $$\begin{aligned} {\textbf{H}}&={\textbf{F}}_{r} \mathbf {\Gamma } {\textbf{F}}^{H}_{P} \end{aligned}$$

where ${\textbf{F}}_{r} \in {\mathbb {C}}^{N_{r} \times N_{rG}}$ is an over complete matrix and each of its columns has a form $\varvec{a}_{r} ( \phi _{i} )$, with $\phi _{i}$ chosen from a pre-discretized grid, $\mathbf {\Gamma } \in {\mathbb {C}}^{N_{rG} \times N_{IG}}$ is a sparse matrix with *Q* non-zero entries.

Then the received signal by the user at the *t*-th time instant is

13 $$\begin{aligned} {y}(t)&= (\varvec{f}^{H}(t) {\textbf{H}}_{c} \varvec{w}(t))s(t) + \epsilon (t) \end{aligned}$$

where $\varvec{f}(t)$ and $\varvec{w}(t)$ are the combining and precoding vectors at the receiver and transmitter respectively, and the cascaded channel ${\textbf{H}}_{c}$ is

14 $$\begin{aligned} {\textbf{H}}_{c}&= {\textbf{H}} \mathbf {\Theta } {\textbf{G}} = {\textbf{F}}_{r} \mathbf {\Gamma } {\textbf{F}}^{H}_{P} \mathbf {\Theta } {\textbf{F}}_{P} \mathbf {\Sigma } {\textbf{F}}^{H}_{L} \nonumber \\&= {\textbf{F}}_{r} \mathbf {\Gamma } \mathbf {\Xi } \mathbf {\Sigma } {\textbf{F}}^{H}_{L} \end{aligned}$$

where $\mathbf {\Xi } = {\textbf{F}}^{H}_{P} \mathbf {\Theta } {\textbf{F}}_{P}$. In addition,

15 $$\begin{aligned} vec(\mathbf {{H}}_{c})&= vec({\textbf{F}}_{r} \mathbf {{\Gamma } \Xi } \mathbf {{\Sigma } F}^{H}_{L} ) \nonumber \\&= ({\textbf{F}}^{*}_{L} \otimes {\textbf{F}}_{r} ) ( \mathbf {\Sigma }^{T} \otimes \mathbf {\Gamma } ) vec(\mathbf {\Xi }) \nonumber \\&= ({\textbf{F}}^{*}_{L} \otimes {\textbf{F}}_{r} ) ( \mathbf {\Sigma }^{T} \otimes \mathbf {\Gamma } ) ({\textbf{F}}^{T}_{P} \odot {\textbf{F}}^{H}_{P} ) v^{*} \end{aligned}$$

where $\odot$ is the Khatri–Rao product. $\bar{\textbf{D}} = {\textbf{F}}^{T}_{P} \odot {\textbf{F}}^{H}_{P} \in {\mathbb {C}}^{N^{2}_{IG} \times N_{IG}}$ is a matrix which contains only $N_{IG}$ distinct rows which are exactly the first $N_{IG}$ rows of matrix ${\bar{\textbf{D}}}$. Then,

16 $$\begin{aligned} vec(\mathbf {{H}}_{c})&= ({\textbf{F}}^{*}_{L} \otimes {\textbf{F}}_{r} ) {\bar{\mathbf {\Lambda }}} {\bar{\textbf{D}}}_{u} v^{*} \end{aligned}$$

where $\bar{\textbf{D}}_{u} = \bar{\textbf{D}} (1:N_{IG},:), {\bar{\mathbf {\Lambda }}}$ is a merged version of $\bar{\textbf{J}} = \mathbf {\Sigma }^{T} \otimes \mathbf {\Gamma }$ that is ${\bar{\mathbf {\Lambda }}} (: i) = \sum _{n \in {\mathcal {Q}}_{i}} \bar{\textbf{J}}(:, n)$, where ${\mathcal {Q}}_{i}$ is a set of all indices associated with those rows of ${\bar{\textbf{D}}}$ that is identical to the *i*-th row of ${\bar{\textbf{D}}}$. So,

17 $$\begin{aligned} vec(\mathbf {{H}}_{c})&= ({\textbf{F}}^{*}_{L} \otimes {\textbf{F}}_{r} ) {\bar{\mathbf {\Lambda }}} {\bar{\textbf{D}}}_{u} v^{*} \nonumber \\&= (({\bar{\textbf{D}}}_{u} v^{*})^{T} \otimes ({\textbf{F}}^{*}_{L} \otimes {\textbf{F}}_{r}) ) vec({\bar{\mathbf {\Lambda }}}) \nonumber \\&= {\textbf{K}} \varvec{\bar{x}} \end{aligned}$$

where ${\textbf{K}} = (({\bar{\textbf{D}}}_{u} v^{*})^{T} \otimes ({\textbf{F}}^{*}_{L} \otimes {\textbf{F}}_{r} ))$ and $\varvec{\bar{x}} = vec({\bar{\mathbf {\Lambda }}})$ is a sparse vector to be estimated.

Assume $s(t)=1$ and denote $\varvec{y} = [y(1) \; {y}(2) \; \dots \; {y}(T)]^{T}$, we have

18 $$\begin{aligned} \varvec{y}&= {\textbf{W}}_{f} {\textbf{K}} \varvec{\bar{x}} + \varvec{\epsilon } \end{aligned}$$

where ${\textbf{W}}_{f} \in {\mathbb {C}}^{T \times N_{t} N_{I}}, {\textbf{W}}_{f}(t,:) = \varvec{w}^{T}(t) \otimes \varvec{f}^{H}(t)$, and ${\textbf{W}}_{f}(t,:)$ is the *t*-th row of ${\textbf{W}}_{f}$.

## Results and discussion

In this chapter, the simulation results of channel estimation techniques are discussed. Three techniques are applied in the estimation process. These are Oracle, conventional least squares (LS), and orthogonal matching pursuit (OMP). The value of $\beta$, which indicates how well the IRS reflects impinging signals, is specified as 1 (perfect reflection), 0.8, 0.5, and 0.2 (poor reflection) for OMP whereas $\beta = 1$ for LS and Oracle. Furthermore, each IRS element is assumed to have the same value of $\beta$ at a time. In the Oracle estimation technique, the positions of the non-zero channel matrix coefficients are assumed known prior to estimation. Since Oracle is the estimator with the best performance, it is taken as a benchmark for the conventional LS and OMP estimation techniques. Normalized mean square error (NMSE) and spectral efficiency (SE) are used as performance metrics. The variables used for evaluation are SNR, pilot length T, and number of reflecting elements $(N_{I})$.

**Figure 2:**
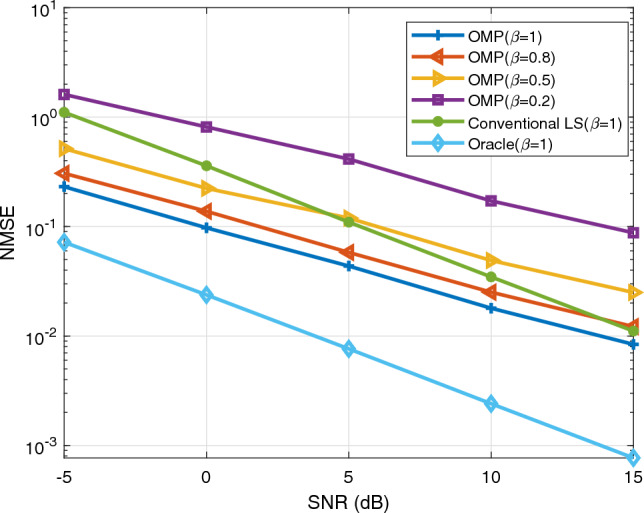
The effect of SNR.

In Fig. 2 the effect of signal-to-noise ratio (SNR) on channel estimation is shown. The number of transmitter antennas, receiver antennas, and IRS elements are kept constant at values 36, 4, and 64 respectively. A pilot length is set to 250 for each of the three estimation techniques. As a small value of $\beta$ means a high imperfection in reflection, NMSE is higher as compared to that when $\beta >0.2$. The conventional LS beats OMP when $\beta =0.2$. It also begins to exceed OMP’s performance at $\beta =0.5$ and SNR value 5 dB. But OMP shows a better performance compared to conventional LS especially when there is a high level of noise (low SNR) and outperforms LS at values of $\beta =1\, \text {and} \, 0.8$. Generally, NMSE decreases as SNR increases.

**Figure 3:**
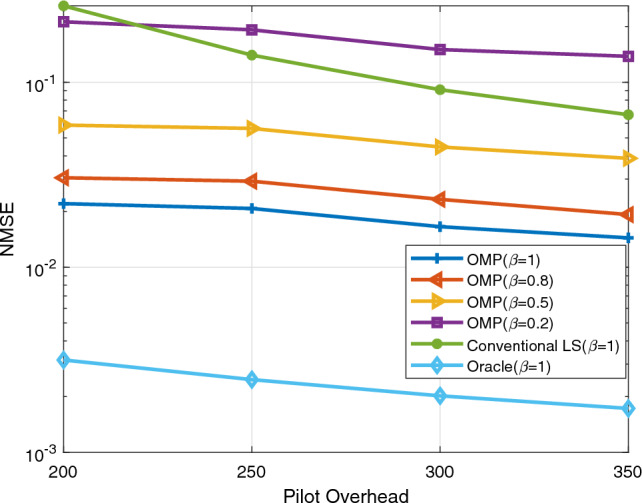
The effect of T.

Figure 3 shows the effect of the length of the pilot signals on the channel estimation process. The values of $N_{t}$, $N_{r}$, and $N_{I}$ are respectively 36, 4, and 64. Moreover, the value of SNR at the transmitter is kept constant at 10 dB. As the value of T increases, the NMSE decreases as expected for all the estimation techniques. This is because enough pilot symbols are available so that better CSI is obtained. The poorest performance is observed for OMP when $\beta =0.2$ i.e., impinging signals are not well reflected so is difficult to get accurate channel state information. The conventional LS is the next to perform poorly. OMP with values of $\beta =1,\, 0.8,\, \text {and}\, 0.5$ beats the LS estimator.

**Figure 4:**
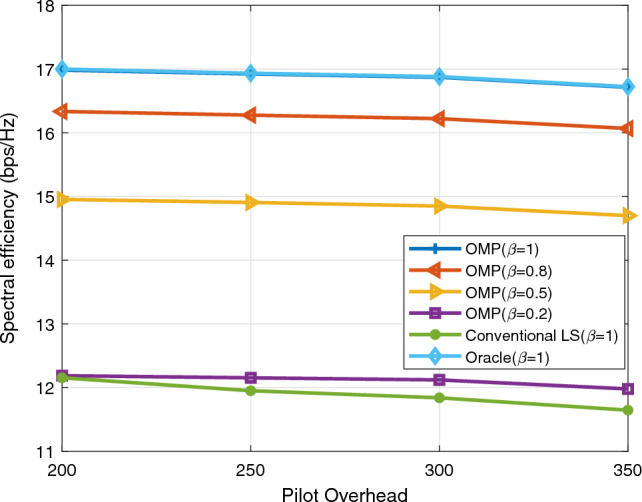
Spectral efficiency as T varies.

The variation of spectral efficiency with T is shown in Fig. 4. The SNR at the transmitter, $N_{t}$, $N_{r}$, and $N_{I}$ are respectively 10 dB, 36, 4, and 64. As T increases the spectral efficiency decreases as expected. The reason for the decrease in spectral efficiency is that as the length of the pilot signal increases the available channel bandwidth for transmission of data symbols decreases.

## Conclusion

In this paper, the channel estimation performance of Oracle, conventional LS, and OMP estimation techniques is evaluated based on NMSE and spectral efficiency. The effect of signal-to-noise ratio (SNR) and the length of the pilot signal (T) is shown. Imperfect reflection of signals impinging on the IRS is also considered by varying the value of $\beta$ as 1 (perfect reflection), 0.8, 0.5, and 0.2 (poor reflection) for the OMP-based channel estimation. For the Oracle, which is the benchmark, and conventional LS, perfect reflection i.e. $\beta$ = 1 is assumed. NMSE decreases as SNR and T increase. The spectral efficiency decreases as T increases as the increase in T decreases the available channel bandwidth which should be used for the transmission of data symbols. Generally, in both of the performance metrics, OMP estimator shows better performance than conventional LS especially at the values of $\beta$ = 1, 0.8, and 0.5.

## Data Availability

The MATLAB codes used during the current study are available from the corresponding author on reasonable request.

## Abbreviations

5G: Fifth generation
LS: Least squares
NMSE: Normalized mean square error
OMP: Orthogonal matching persuit
SE: Spectral efficiency
SNR: Signal to noise ratio
THz: Terahertz
